# Orthodontists’ Perceived Knowledge, Confidence, and Clinical Practices in Pediatric Temporomandibular Disorders

**DOI:** 10.3390/children13040445

**Published:** 2026-03-25

**Authors:** Thomas Southern, Linda Sangalli, Calli A. Marando, Caroline M. Sawicki

**Affiliations:** 1 Adams School of Dentistry, University of North Carolina, Chapel Hill, NC 27599, USA; trsouth@unc.edu; 2 College of Dental Medicine—Illinois, Midwestern University, Downers Grove, IL 60515, USA; lsanga@midwestern.edu; 3 Department of Orthodontics, University of North Carolina, Chapel Hill, NC 27599, USA; cmarando@unc.edu; 4 Department of Pediatric Dentistry and Dental Public Health, University of North Carolina, Chapel Hill, NC 27599, USA

**Keywords:** temporomandibular disorders, pediatric, orthodontists

## Abstract

**Highlights:**

**What are the main findings?**

- Fewer than half of orthodontists reported routinely using validated screening questions for pediatric temporomandibular disorders (TMD) and overall perceived knowledge and confidence were moderate.
- Routine use of structured TMD screening was independently associated with significantly higher perceived knowledge and confidence in screening, diagnosis, and management.

**What is the implication of the main findings?**

- Standardized pediatric TMD screening may function not only as a diagnostic tool but also as an educational scaffold that reinforces clinical reasoning and provider confidence.
- Enhancing orthodontic training and continuing education aligned with the Diagnostic Criteria for TMD pediatric recommendations may reduce variability and support earlier identification and appropriate referral of pediatric TMD.

**Abstract:**

Background/Objectives: Temporomandibular disorders (TMD) are common in pediatric patients, yet limited data exist on orthodontists’ knowledge, confidence, and clinical practices related to pediatric TMD. This cross-sectional study aimed to characterize orthodontists’ perceived knowledge, confidence, training, and practice patterns, and examine associations between routine screening behaviors and perceived confidence. Methods: A 34-item anonymous survey was distributed to orthodontists and orthodontic residents enrolled in or graduated from U.S. Commission on Dental Accreditation (CODA)-accredited programs. The survey assessed perceived knowledge, confidence in screening, diagnosis, and management of pediatric TMD, adequacy of residency training (on 0–10 numerical rating scale), frequency of routine TMD screening and examination practices, and referral patterns. Respondents were compared in study outcomes according to years of clinical practice with ANOVA. Respondents were categorized according to frequency of TMD screening (always/some of the time vs. sometimes/never) and compared in study outcomes using independent *t*-tests. Results: Out of 83 respondents, perceived knowledge (56.8 ± 26.9), confidence with screening (62.0 ± 30.5), diagnosis (59.4 ± 29.8), and management (50.8 ± 30.9) of pediatric TMD were moderate. Less than half of respondents (45.8%) reported routinely screening pediatric patients using standardized screening questions. Orthodontists who reported routine screening demonstrated significantly greater perceived knowledge and confidence in screening, diagnosis, and management compared with those who screened less frequently (all *p*’s ≤ 0.018, effect size between 0.57 and 0.78). Greater use of specific history-taking and clinical examination components was also associated with higher perceived confidence (all *p*’s between 0.001 and 0.046, effect size between 0.53 and 1.01). Confidence differed by years in practice, with lower scores reported among residents and mid-career practitioners (*p* < 0.05). Conclusions: Variability exists in orthodontists’ perceived knowledge, confidence, and clinical practices regarding pediatric TMD. Routine screening was associated with greater perceived competence. These findings highlight potential alignment between structured screening behaviors and self-reported confidence and may inform educational strategies in orthodontic training.

## 1. Introduction

Temporomandibular disorders (TMD) represent a heterogeneous group of conditions affecting the temporomandibular joint (TMJ), masticatory muscles, and associated structures [1]. Although once regarded as an adult condition, TMD is increasingly recognized in child and adolescent populations, with prevalence estimates varying widely, ranging from approximately 4% to over 60% depending on diagnostic criteria, sampling, and age [2,3,4,5,6]. Symptoms often emerge or intensify during adolescence and frequently co-occur with headache, psychosocial distress, and other pain conditions, contributing to functional impairment and reduced quality of life [7,8]. Early identification of pediatric TMD is clinically important to facilitate timely education, conservative management, and appropriate referral.

Pediatric dentists and orthodontists are uniquely positioned to perform routine and repeated screening because they provide long-term care with regular follow-up visits during the same developmental periods in which TMD signs and symptoms commonly emerge. Through routine evaluation of craniofacial growth, occlusion, and jaw function, these providers are well suited to recognize early indicators of TMD in children and adolescents. Despite this strategic position, the recent literature has reported low perceived knowledge and confidence among pediatric dentists regarding TMD diagnosis, screening, and management [9]. However, pediatric dentists with greater perceived knowledge and confidence in TMD were also more likely to report encountering TMD signs and symptoms in up to 50% of their patients, underscoring the role of provider-related behavioral factors in shaping routine clinical practices. Previous studies suggest that both predoctoral and postdoctoral dental training may provide limited formal education on TMD [9,10,11,12], potentially contributing to inconsistent knowledge and confidence among providers. Yet no study to date has specifically investigated how clinical practices regarding TMD vary among orthodontists with respect to screening, assessment, and referral. Moreover, controversies may exist regarding the role of orthodontic treatment in TMD etiology and management, especially among providers with different years of clinical practice.

Significant progress has been made with the recent establishment of Diagnostic Criteria for TMD recommended for the pediatric population, where standardized screening tools, such as brief symptom-based questionnaires and focused clinical examination components, have been recommended to support early detection of TMD in children and adolescents [13,14,15]. These frameworks allow orthodontists to move beyond anecdotal and vague observation toward more routine, systematic assessment, evidence-based diagnosis, and improved communication among treating providers. However, little is known about how frequently orthodontists screen pediatric patients for TMD or whether routine screening is associated with greater perceived knowledge and clinical confidence, as suggested among pediatric dentists.

Therefore, the objectives of this study were to characterize orthodontists’ perceived knowledge, confidence, training, and clinical practices related to pediatric TMD, and to examine associations between routine screening behaviors and perceived competence in screening, diagnosis, and management. We hypothesized that orthodontists reporting greater perceived knowledge and confidence in pediatric TMD would be more likely to routinely screen their patients for signs and symptoms of TMD.

Improved understanding of current practice patterns may inform educational strategies and support the integration of standardized pediatric TMD screening into orthodontic training and clinical care.

## 2. Materials and Methods

Study Design. This cross-sectional survey study was reviewed by the University of North Carolina Institutional Review Board (IRB) and deemed exempt (24-1119, 8 May 2024). An anonymous online questionnaire was administered via Qualtrics (Provo, UT, USA) and distributed through the American Association of Orthodontists (AAO) Partners in Research (PIR) program. In accordance with AAO survey distribution policy, the invitation was sent to a random sample of 2300 active U.S. AAO members. The AAO disseminated the survey invitation twice in June 2025, and reminder emails were distributed to the sampled members during the data collection period. The survey remained open from June 2025 through January 2026. Eligible participants needed to be completing or have already completed specialized training in orthodontics at a U.S. CODA-accredited advanced dental education program. Electronic informed consent was obtained from respondents prior to participation. In accordance with local IRB regulations, participants were not obligated to respond to all survey items.

Survey Assessment Tool. The anonymous survey (Supplementary Materials S1) was adapted from a previously published instrument assessing pediatric dentists’ practice patterns in the screening, diagnosis, and management of TMD [9]. Core domains retained from the original instrument included perceived knowledge and confidence, frequency of screening behaviors, referral patterns, and management approaches.

Modifications were made to align the instrument with orthodontic scope of practice. These included revision of terminology to reflect orthodontic clinical workflows and addition of orthodontic-specific management and decision-making items (e.g., deferring orthodontic treatment, fabrication of occlusal splints, ordering TMJ imaging, and specialty referral patterns). Items assessing orthodontic training background and years in practice were also included.

Prior to dissemination, the adapted survey was reviewed by two orthodontists (L.S. and C.A.M.), one orofacial pain specialist (L.S.), and one pediatric orofacial pain researcher (C.M.S.) with clinical and academic experience to assess item clarity, relevance, and content appropriateness for orthodontic practice. Minor wording refinements were made based on their feedback. Given the multi-domain, descriptive nature of the instrument and the use of single-item measures to assess distinct self-reported perceptions and routine clinical practices, formal psychometric scale validation was not appropriate. Items were analyzed at the individual level and were not intended to measure latent constructs or objective clinical competence. This expert review supported face and content validity of the instrument for the target population. The finalized survey consisted of 34 items organized across multiple sections. Initial five items assessed agreement level (from 0 “strongly disagree” to 100 “strongly agree”) on perceived knowledge of pediatric TMD, confidence in screening, diagnosis, and management, and perceived adequacy of residency training. These items assessed self-reported perceptions and did not include objective testing of knowledge, diagnostic accuracy, or observed clinical performance. Subsequent sections assessed the frequency with which specific TMD-related screening history questions (e.g., 3Q-TMD including pain in TMJ or pre-auricular area, pain with jaw function, and episodes of jaw lock; difficulty in mouth opening; self-reported TMJ noises; change in bite; history of trauma; previous TMD treatments; and self-reported parafunctional activities) and clinical examination components (e.g., muscles and TMJ palpations, TMJ noises auscultation, assessment of mandibular range of motion) were used in routine practice. Responses were recorded using a five-point Likert-type scale with the following options: “always”, “most of the time”, “about half the time”, “sometimes”, and “never”. Additional items assessed the frequency with which respondents routinely encountered patients with TMD in clinical practice and the frequency of TMD referrals. Item also evaluated beliefs regarding malocclusion, orthodontic treatment, and TMD; referral patterns; patient presentation characteristics (including age groups and commonly observed comorbidities); and recommended management strategies. The final section collected information on professional background, years in practice, practice setting, geographic region, and sociodemographic characteristics.

Data Analysis. Descriptive statistics were computed to summarize outcome variables. Perceived knowledge, confidence levels in pediatric TMD screening, diagnosis, and management, and adequacy of training were compared across respondents according to years of clinical practice with analyses of variance (ANOVAs), with Bonferroni as post hoc tests. Next, respondents were categorized according to the frequency of routine TMD screening. Those who reported to always/some of the time performing TMD screening (based on different items of history taking and clinical examination) were assigned a value of 1, while those who reported to sometimes/never conducting such screening were assigned a value of 0. The two groups were compared in overall perceived knowledge, confidence levels in pediatric TMD screening, diagnosis, and management, and perceived adequacy of training with independent *t*-tests. To assess the robustness of the primary dichotomization, a sensitivity analysis was conducted using an alternative grouping of the original frequency responses, combining always, some of the time, and about half the time versus sometimes and never.

To examine independent predictors of perceived general TMD knowledge and confidence in screening, diagnosis, and management, separate multiple linear regression models were constructed for each outcome. Routine use of the three-item questions 3Q/TMD (always/most of the time vs. sometimes/never), years of clinical experience (modeled using dummy variables with residents serving as the reference group), perceived adequacy of TMD training during orthodontic residency, and frequency of pediatric patients presenting with TMD signs and symptoms were entered simultaneously as independent variables. Assumptions of linear regression were evaluated through inspection of residual plots and normal probability plots, and multicollinearity among predictors was assessed using variance inflation factors.

Data analyses were conducted with SPSS (IBM SPSS, v29, IBM Corp., Armonk, NY, USA), and statistical significance was set at 0.05.

## 3. Results

### 3.1. Study Sample

**Figure 1:**
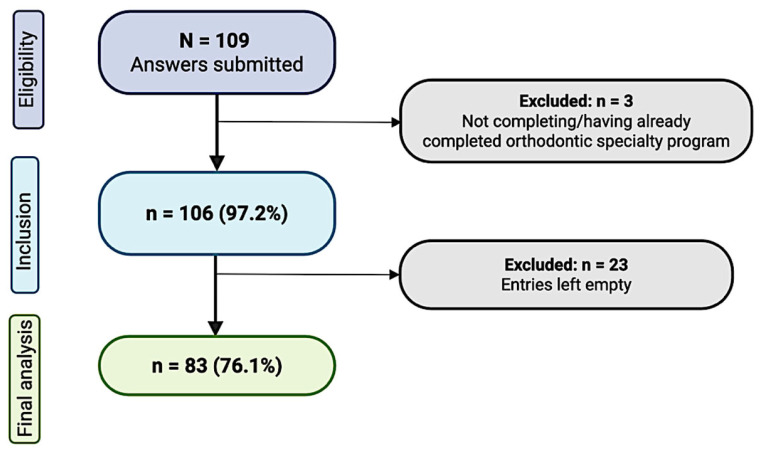
Participant Flowchart.

The survey was distributed to a random sample of 2300 active U.S. members of the AAO in accordance with AAO survey distribution policy. A total of 109 responses were submitted, yielding a response rate of 4.7% (109/2300). Of these, 3 responses were not eligible because the respondents had not completed or were not currently enrolled in a specialized CODA-accredited orthodontic training program. Of the remaining 106 responses, 23 were left empty and excluded. The final analysis included 83 participants, corresponding to a final response rate of 3.6% (83/2300). Participants were 57.8% male, with a mean age of 49.6 ± 16.3 years. The participant flowchart is illustrated in Figure 1, and demographic characteristics are presented in Table 1.

### 3.2. Perceived Knowledge, Confidence, and Training Related to Pediatric TMD

Overall, respondents reported moderate levels of perceived knowledge about pediatric TMD (56.8 ± 26.9), comfort with screening (62.0 ± 30.5), diagnosis (59.4 ± 29.8), and management (50.8 ± 30.9) of TMD in pediatric patients. Perceived adequacy of TMD training during orthodontic residency was also rated as moderate (50.1 ± 28.3).

### 3.3. Frequency of TMD Screening History and Clinical Examination

**Figure 2:**
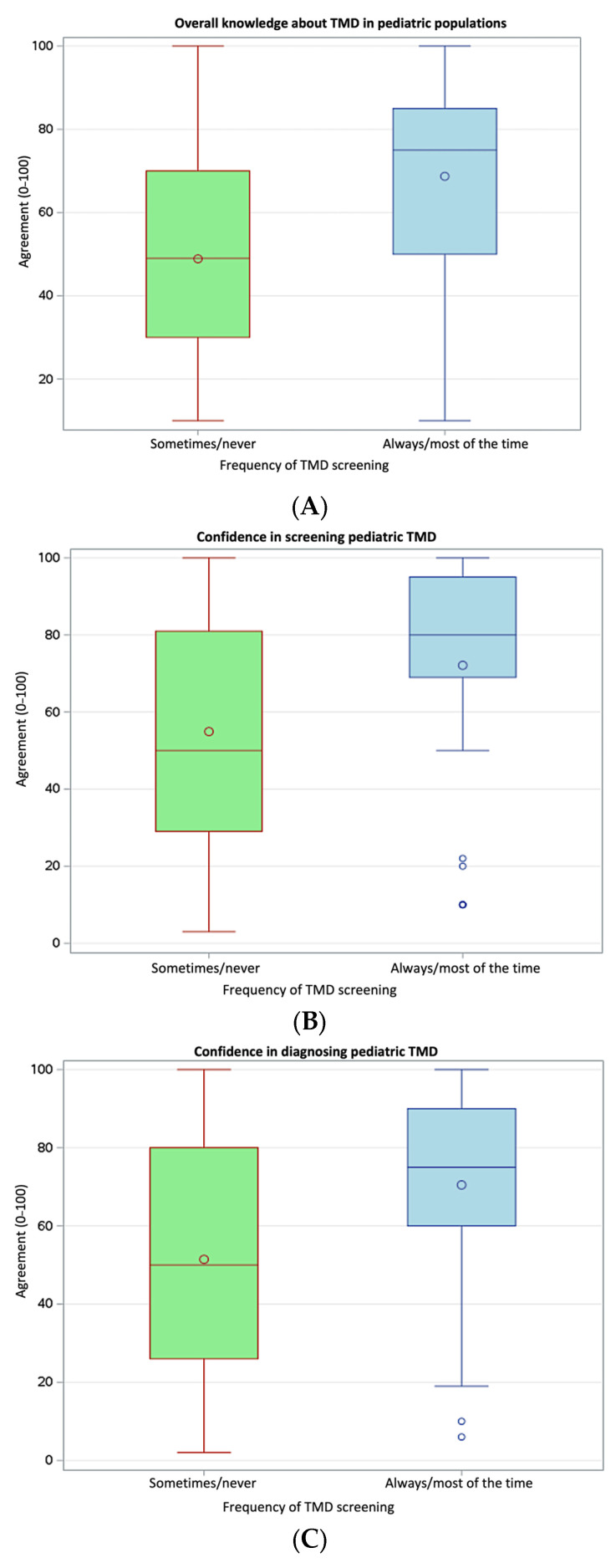

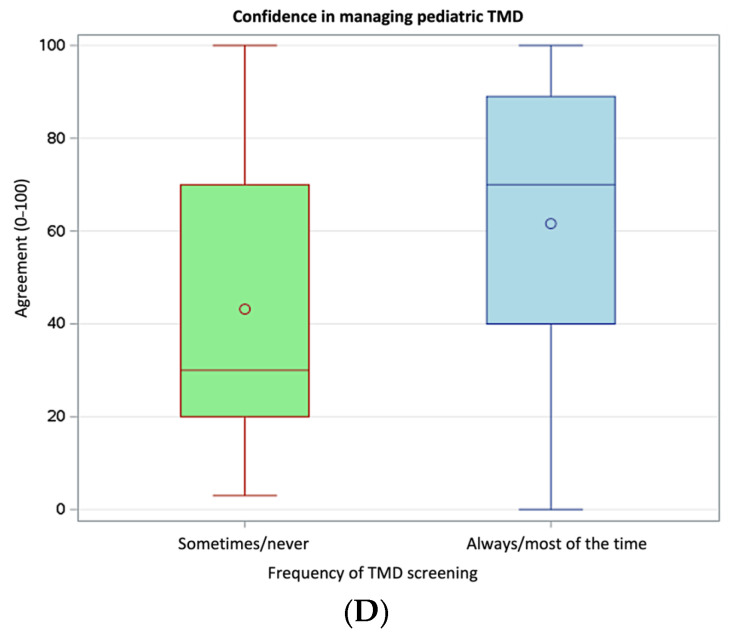
Comparison in perceived knowledge (**A**) and confidence levels in screening (**B**), diagnosis (**C**), and management (**D**) in pediatric TMD according to frequency of routine TMD screening.

Nearly half of the respondents (45.8%) reported always or most of the time routinely screening pediatric patients using the item questions of the validated three screening questions for TMD (3Q-TMD) [16,17]. Compared with respondents who reported sometimes or never using the 3Q-TMD, those who screened always or most of the time reported significantly higher perceived general knowledge about TMD (68.7 ± 23.6 vs. 48.9 ± 26.9, *p* = 0.002, Cohen’s *d* = 0.78, Figure 2A) and greater confidence in screening (72.1 ± 28.7 vs. 54.9 ± 31.0, *p* = 0.018, Cohen’s *d* = 0.57, Figure 2B), diagnosis (70.5 ± 27.3 vs. 51.4 ± 30.7, *p* = 0.007, Cohen’s *d* = 0.65, Figure 2C), and management (61.6 ± 29.6 vs. 43.2 ± 30.9, *p* = 0.012, Cohen’s *d* = 0.61, Figure 2D) of pediatric TMD. No significant difference was observed in perceived adequacy of residency training (*p* = 0.098). Similar direction and statistical significance of the results were obtained by combining respondents who reported always, most of the time, and about half the time compared to those who screened sometimes and never.

Among screening history items, 62.7% of respondents reported always or most of the time inquiring about pain in the ears or cheeks and pain with jaw function, 68.7% reported routinely asking about TMJ noises, and 44.6% reported routinely asking about episodes of jaw locking. With respect to clinical examination, respondents reported always or most of the time masticatory muscle palpation (60.2%), TMJ palpation (67.5%), and assessment of mandibular range of motion (62.7%). Only 31.3% reported routinely auscultating the TMJ for joint noises. Frequencies of all screening and examination items are detailed in Table 2.

### 3.4. TMD Assessment and Perceived Knowledge and Confidence

Respondents who reported always/most of the time performing most TMD-related screening history items (including difficulty in mouth opening, TMJ noises, pain in the TMJ or preauricular area, pain with jaw function, change in bite, and episodes of jaw locking) reported significantly higher perceived knowledge, confidence in screening, diagnosis, and management, as well as perceived adequacy of training, compared with those who reported sometimes/never performing this history taking (all *p*’s between <0.001 and 0.046), with moderate to large effect sizes (Cohen’s *d* between 0.53 and 1.01, Table 2). Fewer statistically significant differences were observed for assessment of history of trauma or prior treatment for TMD, and no significant differences were found for any outcome based on the frequency of screening for parafunctional activities.

Within the clinical examination domain, respondents who reported always/most of the time performing masticatory muscle palpation reported significantly higher confidence in screening compared with those who reported sometimes/never (*p* = 0.020, Cohen’s *d* = 0.58). Respondents who reported frequently performing TMJ palpation reported significantly higher confidence in screening (*p* = 0.024, Cohen’s *d* = 0.61), diagnosis (*p* = 0.027, Cohen’s *d* = 0.60), and management (*p* = 0.035, Cohen’s *d* = 0.57). Respondents who reported frequently assessing mandibular range of motion reported higher confidence in management only (*p* = 0.031, Cohen’s *d* = 0.58), whereas frequency of TMJ noises auscultation showed no significant differences across any outcome domains (Table 2). Sensitivity analyses were conducted using an alternative dichotomization of the ordinal frequency scale, grouping always, most of the time, and about half of the time versus sometimes and never. The direction and statistical significance of the results were unchanged, except for few items highlighted in the additional analysis (Supplementary Materials Table S1).

Perceived knowledge and confidence in screening, diagnosis, and management of pediatric TMD significantly differed according to years in practice. Respondents with more than 10 years of clinical experience and those 0–2 years post-residency reported similarly higher scores across all domains, whereas respondents currently enrolled in residency and those practicing 5–10 years post-residency reported lower scores. This pattern was consistent across all assessed domains (This should be Supplementary Materials Table S2).

A series of linear regression analyses were conducted to examine independent predictors of perceived general TMD knowledge, confidence in screening, diagnosis, and management. The model explained 39.4% of the variance in perceived knowledge of pediatric TMD (adjusted R^2^ = 0.39, *p* < 0.001). Higher perceived adequacy of residency training (B = 0.4 per one-point increase, *p* < 0.001, 95% CI 0.2, 0.6) and over 10 years of practice (B = 15.9, *p* = 0.011, 95% CI 3.8, 27.9) were independently associated with greater perceived knowledge. The models explained between 28.3% and 38.0% of the variance in reported confidence in TMD screening, diagnosis, and management. Across all models, higher perceived adequacy of residency training (*p* < 0.001) and over 10 years of practice (*p’s* between 0.006 and 0.008, respectively) were independently associated with greater perceived confidence in TMD screening and diagnosis. (Table 3).

### 3.5. Malocclusions Associated with TMD

**Figure 3:**
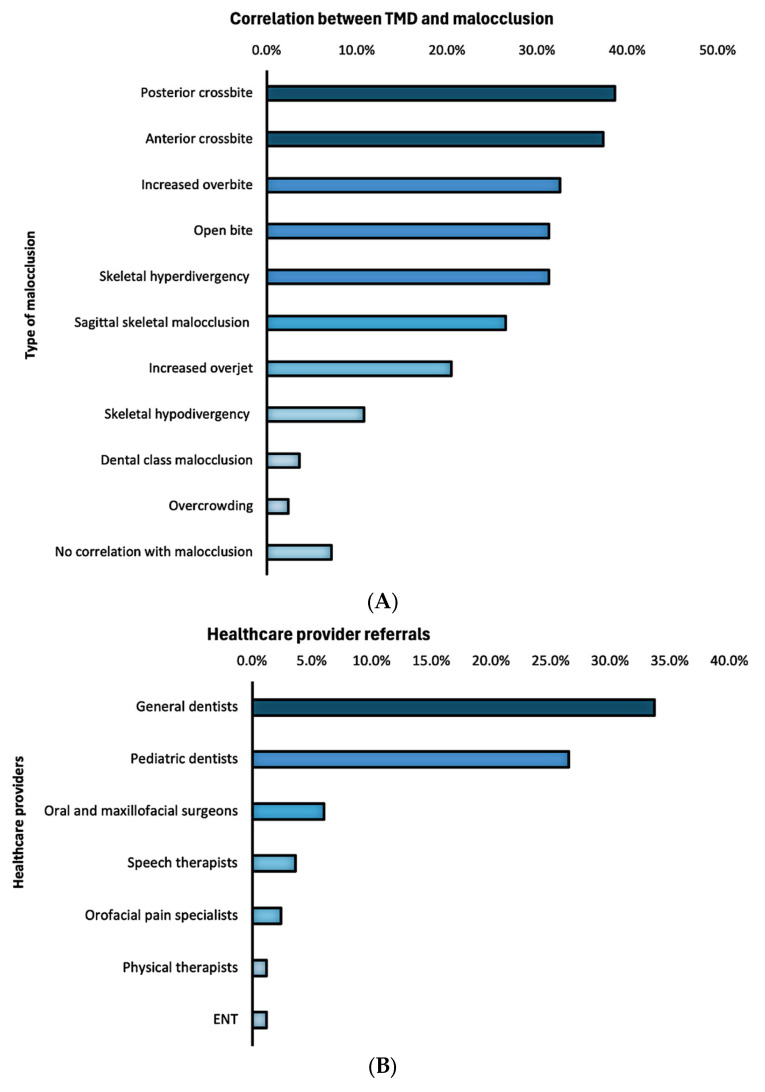
Types of malocclusions most frequently correlated with TMD (**A**) and healthcare providers providing referrals (**B**).

The respondents reported moderate confidence that malocclusion is one of the contributing factors to TMD (42.2 ± 35.1). Lower confidence was reported about the statements that orthodontic treatment can treat TMD (32.3 ± 32.0) and that orthodontic treatment causes TMD (9.4 ± 14.7). Respondents reported several malocclusion types as being commonly associated with TMD, most frequently posterior crossbite (38.6%), anterior crossbite (37.3%), increased overbite (32.5%), open bite (31.3%), skeletal hyperdivergency (31.3%), and sagittal skeletal malocclusion (26.5%), among others (Figure 3A). Low-to-moderate agreement was reported about the statement that orthodontic treatment has no relationship with TMD (37.3 ± 35.1).

### 3.6. Referral Patterns, Patient Presentation, and Management

Nearly half of respondents (48.0%) reported to be referred up to 25% of pediatric patients for TMD, whereas 50.7% reported not to be referred any pediatric patients for TMD. Referrals primarily originated from general dentists (33.7%) and pediatric dentists (26.5%), among other providers (Figure 3B).

**Figure 4:**
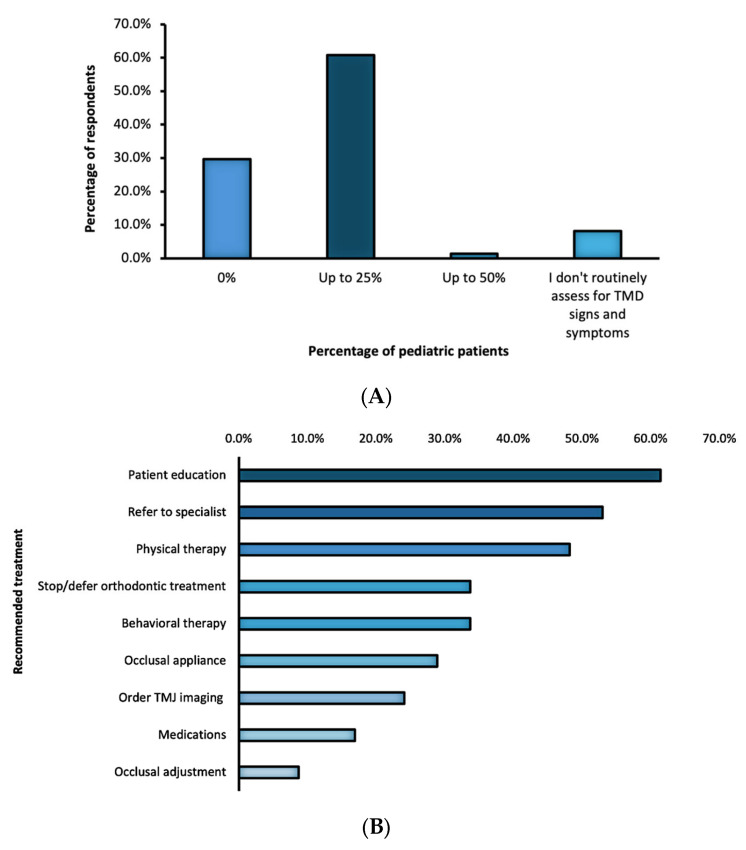

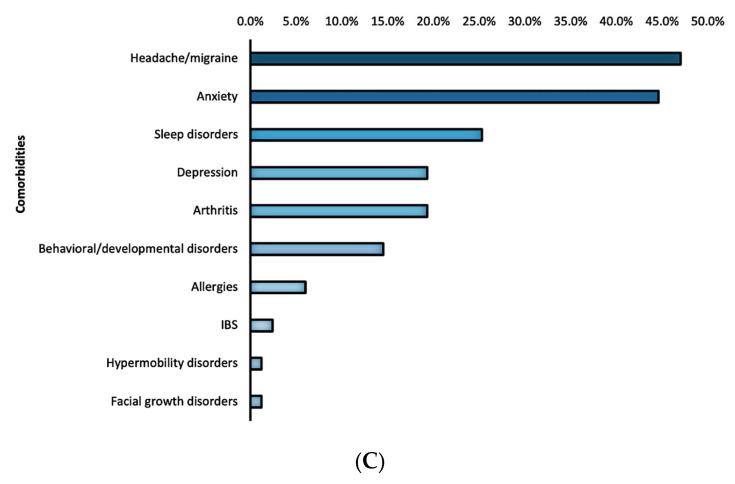
Frequency distribution of pediatric patients presenting with TMD signs and/or symptoms per week (**A**), recommended treatments for these patients (**B**) and comorbidities observed in patients with TMD (**C**).

The majority of respondents (60.8%) reported that up to 25% of pediatric patients presented weekly with TMD signs and/or symptoms (Figure 4A). These patients were most commonly adolescents aged 15–18 (50.6%) and 12–14 years (49.4%), with lower frequencies reported among children aged 9–11 years (8.4%) and 6–8 years (1.2%). When pediatric patients present with TMD signs and/or symptoms, respondents most commonly reported providing patient education (61.4%), referring to a specialist (53.0%), recommending physical therapy (48.2%), deferring orthodontic treatment (33.7%), and recommending behavioral therapy (33.7%). Referrals were most frequently to an orofacial pain specialist (37.3%), followed by oral and maxillofacial surgeons (25.3%), physical therapists (10.8%), psychologists (1.2%), or general dentists with focus on TMD (1.2%). Additional management strategies included ordering TMJ imaging (24.1%), delivering an occlusal appliance (28.9%), prescribing medications (16.9%), and performing occlusal adjustment (8.7%, Figure 4B).

Respondents reported several comorbidities as being commonly observed in pediatric patients with TMD, most frequently headache/migraine (47.0%), anxiety (44.6%), sleep disorders (25.3%), depression (19.3%), and arthritis (19.3%). Less commonly reported comorbidities included behavioral/developmental disorders (14.5%), allergies (6.0%), and irritable bowel syndrome (2.4%, Figure 4C).

## 4. Discussion

Orthodontists occupy a pivotal role in identifying TMD in younger populations. The objectives of this study were to characterize orthodontists’ perceived knowledge, confidence, training, and clinical practices related to pediatric TMD, and to examine whether routine screening behaviors were associated with perceived competence in screening, diagnosis, and management. In this cohort, orthodontists reported overall moderate levels of perceived knowledge, confidence, and adequacy of training, with considerable variability across respondents. Notably, fewer than half reported routinely using validated symptom-based screening questions, and routine screening behaviors were consistently associated with significantly greater perceived knowledge and confidence across domains. These findings highlight variability in current practice patterns and suggest an association between structured screening behaviors and higher self-reported competence.

### 4.1. Variability in Perceived Knowledge, Confidence, and Training

The moderate levels of perceived knowledge and confidence observed in this study are consistent with prior research demonstrating heterogeneity in TMD education across dental training programs. National surveys of predoctoral curricula have shown wide variation in didactic hours, clinical exposure, and use of standardized diagnostic criteria for TMD, with many programs reporting limited structured or clinical instruction [10]. Similar variability has been documented within orthodontic and pediatric dentistry postgraduate programs, where TMD education is often integrated inconsistently into broader curricula [18]. Collectively, these findings suggest that orthodontists may enter practice with differing levels of preparation in pediatric TMD assessment and management, potentially contributing to variability in confidence and screening behaviors.

Importantly, perceived adequacy of residency training in our cohort was rated as only moderate and did not significantly differ between routine screeners and non-screeners. This may indicate that clinical behaviors are shaped not only by formal residency training, but also by post-graduate experiences, continuing education, mentorship, and practice environment. It also highlights the need for clearer curricular benchmarks aligned with contemporary diagnostic frameworks such as the DC/TMD pediatric adaptations [13,14,15].

### 4.2. Routine Screening and Structured Assessment

One of the most clinically meaningful findings of this study was the consistent association between higher perceived competence and routine TMD screening behaviors, with regression analyses indicating that structured screening was marginally associated with higher perceived knowledge scores. Orthodontists who reported routinely asking validated symptom-based questions, such as the 3Q/TMD screening tool, demonstrated significantly higher perceived knowledge and confidence in screening, diagnosis, and management. The 3Q/TMD has demonstrated acceptable validity relative to the DC/TMD in adults and reliability in adolescent populations [16,17]. While screening tools are not diagnostic instruments, they provide a structured method for identifying patients who may require further evaluation. The observed association between structured screening and perceived competence is consistent with prior dental education research demonstrating that greater clinical exposure and structured TMD instruction are associated with higher knowledge and confidence among trainees [10].

Moreover, respondents who routinely assessed core symptom domains aligned with DC/TMD recommendations, including pain in the preauricular area, pain with jaw function, joint noises, and locking episodes, reported greater perceived competence across multiple domains. These findings suggest that orthodontists who report integrating standardized history-taking and examination components also report greater perceived competence. Structured assessment may reduce ambiguity, promote systematic evaluation, and improve interdisciplinary communication.

Interestingly, TMJ auscultation was not associated with greater perceived competence, whereas palpation of masticatory muscles and TMJ structures, as well as range-of-motion assessment, were more strongly associated with confidence. This pattern is consistent with DC/TMD diagnostic principles, which prioritize pain-related findings and functional limitations over isolated joint sounds [15]. Studies indicate that up to approximately one-quarter to one-third of healthy adolescents may exhibit audible joint sounds without pain or dysfunction [19]. Thus, symptom-free clicking or mild deviation should not automatically prompt a diagnosis of internal derangement. This may explain the lack of association observed in the current study.

### 4.3. Experience and Confidence Across Career Stages

The non-linear relationship between years in practice and confidence warrants consideration. Residents and mid-career orthodontists (5–10 years post-residency) reported lower perceived competence compared with early-career (0–2 years post-residency) and highly experienced clinicians (>10 years). Multiple regression analyses further indicated that over 10 years of clinical practice and higher perceived adequacy of residency training were independently associated with higher perceived knowledge and confidence across all domains of pediatric TMD care. Early-career orthodontists may benefit from recent exposure to contemporary diagnostic frameworks during training, while highly experienced practitioners may rely on accumulated clinical experience and established referral networks. Mid-career practitioners may represent a transitional group whose training preceded newer diagnostic updates, but who have not yet accumulated decades of clinical exposure. This pattern highlights the importance of accessible continuing education focused on pediatric TMD assessment and interdisciplinary management. As diagnostic frameworks evolve, particularly with the publication of the DC/TMD pediatric adaptations [13,14,15], ongoing professional development becomes essential to maintain alignment with current standards.

Given that greater years in practice were independently associated with higher perceived competence, and that the sample was predominantly composed of orthodontists with more than 10 years of clinical experience, the overall perceived knowledge and confidence estimates reported in this study may reflect the perspectives of more experienced practitioners. Although years in practice were included in regression models, the distribution of respondents may limit the extent to which findings generalize to residents and early-career orthodontists.

### 4.4. Malocclusion, Orthodontic Treatment, and TMD Beliefs

Respondents reported low confidence that orthodontic treatment causes TMD and only moderate belief that malocclusion is a contributing factor. These findings are generally aligned with contemporary evidence indicating that orthodontic treatment neither causes nor prevents TMD and that occlusal factors alone play a limited etiologic role [20]. Epidemiological research indicates that the prevalence of TMD signs, symptoms, and related functional impairment increases from childhood to adolescence [7,8], a trend associated with hormonal [21] pubertal changes, heightened psychological distress [22,23], stress-related parafunctional activities [24], and greater pain awareness [22]. Thus, the mere co-occurrence of TMD during orthodontic treatment has at times been attributed to the orthodontic intervention itself. However, this temporal association may be more appropriately explained by the natural history of TMD and statistical phenomena such as regression to the mean, rather than a direct causal effect of orthodontic therapy [20].

Although specific malocclusions such as posterior crossbite, open bite, and skeletal discrepancies were commonly perceived as associated with TMD, epidemiologic studies in pediatric populations have demonstrated weak or inconsistent associations between occlusal traits and TMD pain [2,25]. The persistence of moderate beliefs regarding occlusal contribution may reflect historical paradigms within orthodontics or clinical observations of functional discrepancies, highlighting the need for continued emphasis on the multifactorial model of TMD in orthodontic education.

### 4.5. Referral Patterns and Comorbidities

Nearly half of respondents reported referring pediatric patients with TMD, most frequently to orofacial pain specialists and oral and maxillofacial surgeons. Referral to orofacial pain specialists aligns with contemporary recommendations emphasizing conservative, reversible, and interdisciplinary management approaches for pediatric TMD [13,14,15]. Referral to oral and maxillofacial surgeons may reflect cases involving structural joint pathology, diagnostic uncertainty, or more complex presentations requiring surgical consultation rather than routine surgical intervention. The relatively lower rates of referral to behavioral health providers may represent an opportunity for further integration of psychosocial assessment and management strategies, particularly given the high prevalence of anxiety, depression, and other psychosocial comorbidities in pediatric TMD populations [22,23].

The comorbidities most commonly reported in this study, including headache/migraine, anxiety, sleep disorders, and depression, are well documented in epidemiologic studies of adolescents with TMD pain [26,27,28,29]. TMD frequently co-occurs with other pain conditions and psychosocial distress, contributing to functional impairment and reduced quality of life [30]. The recognition of these comorbidities among respondents suggests awareness of the broader biopsychosocial context, though formalized screening for these domains was not assessed in the present study.

### 4.6. Clinical and Educational Implications

The findings of this study have several important implications. First, incorporation of brief, validated screening tools such as the 3Q/TMD into routine orthodontic assessments may facilitate earlier identification of symptomatic patients. Second, alignment of orthodontic training programs with DC/TMD pediatric recommendations could reduce variability in screening and examination practices. Third, targeted continuing education, especially for mid-career orthodontists, may enhance confidence and standardize care. Importantly, the observed association between structured screening and perceived competence raises the possibility that standardized frameworks may be linked to greater familiarity and confidence. However, given the cross-sectional design and reliance on self-reported measures, causality cannot be inferred, and it is equally plausible that clinicians with greater perceived knowledge and confidence are more likely to adopt structured screening practices. In practical terms, standardized screening tools such as the 3Q/TMD could be incorporated into routine orthodontic workflows in several ways. The three screening questions may be embedded into initial patient intake forms, digital health questionnaires, or electronic health record templates completed prior to the first consultation. Positive responses could trigger structured follow-up questions or standardized examination components (e.g., TMJ palpation, range of motion assessment) within institutional protocols. Practices may also incorporate screening prompts into new patient evaluation checklists to ensure consistent implementation across providers. Such integration requires minimal additional chair time and may support systematic identification of symptomatic patients during orthodontic treatment planning.

At the residency level, more structured integration of pediatric TMD education may be warranted. Programs could consider incorporation of standardized screening training (e.g., routine use of the 3Q/TMD) within initial patient evaluation protocols, dedicated didactic modules aligned with DC/TMD pediatric adaptations, and case-based discussions emphasizing diagnostic reasoning and interdisciplinary referral pathways. Competency-based assessment, such as structured clinical case evaluations or simulation exercises, may further support consistent training and reinforce application of standardized screening and examination components. Establishing clearer curricular benchmarks may help reduce variability in preparation across orthodontic programs.

Practice setting may also influence adoption of standardized screening practices. Orthodontists practicing in academic institutions may have greater exposure to evolving diagnostic frameworks, interdisciplinary collaboration, and resident education environments, whereas private practitioners may rely more heavily on continuing education and peer networks. The present study was not powered to detect setting-specific differences; however, future research should examine whether institutional structure, mentorship opportunities, and access to specialty referral networks influence adherence to pediatric TMD screening recommendations.

In summary, practical implementation may begin with incorporating brief validated screening questions into routine intake workflows, reinforcing structured examination components during orthodontic assessments, and aligning both residency curricula and continuing education with DC/TMD pediatric recommendations. Even incremental standardization across these domains may reduce variability in practice and support more consistent identification and management of pediatric TMD.

### 4.7. Strengths and Limitations

This study represents one of the first national surveys to examine orthodontists’ perceived knowledge, confidence, and clinical practices related to pediatric TMD, and it explored clinically meaningful associations between routine screening behaviors and perceived competence. However, several limitations should be considered. The response rate was 3.6%, and although comparable to other survey-based studies distributed through professional society listservs [9,31], this response rate limits generalizability and introduces the potential for nonresponse bias. Some estimates in the linear regression models, particularly for dummy-coded years of clinical experience, may be unstable due to small sample sizes in certain categories. These wide confidence intervals should be interpreted with caution. Moreover, potential interaction effects were not assessed due to sample size limitations, and future studies with larger cohorts may explore these interactions. Because participation was voluntary, orthodontists with greater interest, training, or clinical engagement in TMD may have been more likely to respond, potentially resulting in overrepresentation of clinicians with heightened awareness or confidence in pediatric TMD assessment and management. Conversely, clinicians with lower familiarity or comfort may have been less likely to participate. As such, findings should be interpreted as reflecting the perspectives of respondents rather than the entire U.S. orthodontic population. The cross-sectional design precludes causal inference, and all measures were self-reported, which may not reflect actual clinical behavior or objective competence. Self-reported survey responses are also subject to social desirability bias, whereby respondents may overreport behaviors perceived as professionally appropriate (e.g., routine TMD screening) or overestimate their knowledge and confidence. As a result, reported screening frequencies and perceived competence levels may not fully reflect actual clinical practice. Additionally, knowledge and confidence were assessed through self-reported perceptions rather than objective measures such as standardized knowledge testing, case-based assessment, or observed clinical performance. Perceived knowledge and confidence may not accurately reflect actual clinical competence. Furthermore, the cross-sectional design does not permit determination of directionality; clinicians with greater perceived knowledge and confidence may be more likely to adopt structured screening practices rather than screening leading to increased competence. Future research should incorporate objective assessments of clinical competence, such as standardized case-based evaluations, simulated patient scenarios, or chart-based audits, in combination with survey data to more comprehensively evaluate diagnostic reasoning and management decision-making in pediatric TMD. Prospective longitudinal and interventional studies are particularly needed to assess whether structured educational initiatives, such as targeted residency modules, continuing education programs, or implementation of standardized screening protocols, lead to sustained improvements in knowledge, confidence, and measurable clinical behaviors over time. Such designs would clarify the durability and real-world impact of educational interventions on pediatric TMD care.

## Figures and Tables

**Table 1:** Demographics of the Respondents.

Variable	Answer Options	Frequency (%)
Sex	Male	48 (57.8)
	Female	25 (30.1)
	Prefer Not to Answer	1 (1.2)
	Missing	9 (10.8)
	Hispanic or Latinx	1 (1.2)
	Not Hispanic or Latinx	68 (81.9)
Ethnicity	Unsure	1 (1.2%)
	Prefer Not to Answer	4 (4.8)
	Missing	9 (10.8)
	White	63 (75.9)
	Black or African American	1 (1.2)
	American Indian or Alaska Native	1 (1.2)
Race	Asian	4 (4.8)
	Other	1 (1.2)
	Prefer Not to Answer	4 (4.8)
	Missing	9 (10.8)
	Currently in orthodontic residency	11 (13.3)
	0–2 years post residency	4 (4.8)
Years of Practice	2–5 years post residency	5 (6.0)
	5–10 years post residency	7 (8.4)
	10+ years post residency	47 (56.6)
	Missing	9 (10.8)
	Private practice	41 (49.4)
Clinical Setting	Community clinic or FQHC ^1^	2 (2.4)
	Hospital	2 (2.4)
	Academic institution	25 (30.1)
	Other	4 (4.8)
	Missing	9 (10.8)
	Northeast	10 (12.0)
	Midwest	7 (8.4)
Region of Practice	West	11 (13.3)
	Southeast	39 (47.0)
	Southwest	7 (8.4)
	Missing	9 (10.8)
	General Practice Residency	7 (8.4)
	AEGD	3 (3.6)
	Orofacial Pain	3 (3.6)
Additional Training	Pediatric Dentistry	4 (4.8)
	Dental Sleep Medicine	1 (1.2)
	Physician Assistant	1 (1.2)
	Craniofacial Orthodontics	2 (2.4)
	Other	4 (4.8)

^1^ FQHC: Federally qualified health center; AEGD: Advanced education in general dentistry.

**Table 2:** Perceived knowledge and confidence levels regarding TMD in pediatric populations by frequency of TMD assessment during screening history and clinical examination.

Screening Assessment Item	Frequency of Screening Assessment	Perceived Knowledge About TMD	Confidence in Screening for TMD	Confidence in Diagnosis of TMD	Confidence in Management of TMD	Sufficient Training in TMD During Orthodontic Residency
**Screening History**						
**Difficulty in mouth opening**	Always/most of the time (n = 52)	64.3 ± 25.6	69.0 ± 28.8	67.0 ± 27.1	59.1 ± 29.2	55.9 ± 39.4
	Sometimes/never (n = 24)	41.5 ± 25.7	47.4 ± 31.4	44.2 ± 32.4	35.7 ± 31.3	39.4 ± 24.0
	***p*** ** value (effect size)**	**<0.001 (0.89)**	**0.004 (0.73)**	**0.002 (0.79)**	**0.002 (0.79)**	**0.022 (0.58)**
**TMJ noise**	Always/most of the time (n = 57)	62.8 ± 26.4	68.8 ± 28.4	66.1 ± 27.8	58.8 ± 28.9	55.1 ± 29.3
	Sometimes/never (n = 20)	38.9 ± 21.0	41.8 ± 28.5	39.8 ± 28.0	28.4 ± 26.0	36.8 ± 22.7
	***p* value (effect size)**	**<0.001 (0.95)**	**<0.001 (0.95)**	**<0.001 (0.94)**	**<0.001 (1.01)**	**0.014 (0.66)**
Pain in TMJ or pre-auricular area *	Always/most of the time (n = 52)	64.4 ± 24.1	69.4 ± 27.3	66.6 ± 27.0	59.6 ± 27.9	57.1 ± 28.7
	Sometimes/never (n = 24)	41.8 ± 25.3	47.0 ± 31.8	44.6 ± 31.3	33.7 ± 30.1	36.4 ± 24.0
	***p*** ** value (effect size)**	**<0.001 (0.93)**	**0.002 (0.78)**	**0.002 (0.77)**	**<0.001 (0.91)**	**0.003 (0.76)**
Pain with jaw function *	Always/most of the time (n = 52)	62.7 ± 25.4	67.7 ± 28.1	65.2 ± 27.5	55.7 ± 29.3	55.4 ± 28.6
	Sometimes/never (n = 23)	43.1 ± 27.9	47.1 ± 32.8	45.2 ± 32.8	38.8 ± 33.5	40.6 ± 27.7
	***p*** ** value (effect size)**	**0.004 (0.75)**	**0.007 (0.70)**	**0.008 (0.69)**	**0.031 (0.55)**	**0.040 (0.52)**
**Change in bite**	Always/most of the time (n = 28)	68.5 ± 24.2	71.5 ± 27.9	69.7 ± 28.2	64.9 ± 27.2	59.0 ± 28.9
	Sometimes/never (n = 40)	47.7 ± 27.1	55.3 ± 32.1	52.1 ± 30.6	41.3 ± 30.6	47.6 ± 29.0
	***p*** ** value (effect size)**	**0.002 (0.80)**	**0.035 (0.53)**	**0.018 (0.60)**	**0.002 (0.81)**	0.115 (0.39)
Episodes of jaw lock *	Always/most of the time (n = 37)	68.8 ± 24.2	72.7 ± 28.6	70.1 ± 27.0	62.8 ± 29.4	56.9 ± 31.1
	Sometimes/never (n = 32)	49.4 ± 25.8	55.7 ± 30.5	52.3 ± 30.7	43.0 ± 30.6	47.0 ± 27.3
	***p*** ** value (effect size)**	**0.002 (0.77)**	**0.020 (0.58)**	**0.011 (0.63)**	**0.008 (0.66)**	0.169 (0.34)
**Previous injuries to jaw, head, neck**	Always/most of the time (n = 58)	59.8 ± 26.3	65.2 ± 28.8	62.7 ± 28.6	55.2 ± 29.8	55.3 ± 28.4
	Sometimes/never (n = 19)	47.1 ± 28.7	51.4 ± 34.4	49.2 ± 33.3	38.2 ± 32.8	40.1 ± 28.0
	***p*** ** value (effect size)**	0.076 (0.48)	0.088 (0.46)	0.090 (0.46)	**0.038 (0.56)**	**0.046 (0.54)**
**Previous treatment for TMD**	Always/most of the time (n = 18)	71.8 ± 21.1	72.8 ± 26.8	72.8 ± 25.2	66.6 ± 28.2	58.4 ± 32.5
	Sometimes/never (n = 59)	52.1 ± 27.4	58.6 ± 31.3	55.3 ± 30.6	46.2 ± 30.8	49.0 ± 28.0
	***p*** ** value (effect size)**	**0.006 (0.76)**	0.085 (0.47)	**0.030 (0.60)**	**0.014 (0.68)**	0.233 (0.32)
**Parafunctional activities**	Always/most of the time (n = 45)	59.0 ± 28.9	61.6 ± 31.9	60.8 ± 31.4	54.4 ± 32.5	51.2 ± 28.9
	Sometimes/never (n = 24)	49.5 ± 26.0	60.5 ± 31.5	55.8 ± 31.3	43.9 ± 30.2	50.3 ± 28.8
	***p*** ** value (effect size)**	0.181 (0.34)	0.891 (0.04)	0.533 (0.16)	0.195 (0.33)	0.899 (0.03)
**Clinical Assessment**						
**Masticatory muscle palpation**	Always/most of the time (n = 50)	61.5 ± 25.7	68.3 ± 29.7	66.1 ± 27.7	58.3 ± 27.8	54.3 ± 30.3
	Sometimes/never (n = 25)	48.0 ± 29.9	50.6 ± 31.6	46.5 ± 32.7	38.7 ± 33.7	44.9 ± 26.5
	***p*** ** value (effect size)**	**0.047 (0.49)**	**0.020 (0.58)**	**0.008 (0.67)**	**0.010 (0.65)**	0.176 (0.32)
**TMJ palpation**	Always/most of the time (n = 56)	60.1 ± 26.1	67.1 ± 29.0	64.1 ± 28.1	56.3 ± 29.1	54.0 ± 29.6
	Sometimes/never (n = 19)	47.9 ± 31.0	48.5 ± 34.4	46.2 ± 34.8	38.6 ± 36.0	42.8 ± 27.3
	***p*** ** value (effect size)**	0.100 (0.44)	**0.024 (0.61)**	**0.027 (0.60)**	**0.035 (0.57)**	0.151 (0.39)
**TMJ noise auscultation**	Always/most of the time (n = 26)	59.1 ± 30.1	66.7 ± 31.1	65.4 ± 31.5	59.4 ± 32.3	51.4 ± 31.3
	Sometimes/never (n = 49)	56.0 ± 26.7	61.0 ± 30.8	57.7 ± 29.6	48.0 ± 30.7	51.9 ± 28.7
	***p*** ** value (effect size)**	0.646 (0.11)	0.457 (0.18)	0.298 (0.25)	0.139 (0.36)	0.946 (0.02)
**Mandibular range of motion**	Always/most of the time (n = 52)	60.6 ± 26.5	67.2 ± 29.7	64.3 ± 27.7	56.7 ± 28.8	55.4 ± 30.5
	Sometimes/never (n = 20)	48.9 ± 29.5	51.9 ± 32.0	49.6 ± 34.7	38.8 ± 35.8	42.3 ± 25.2
	***p*** ** value (effect size)**	0.106 (0.43)	0.059 (0.50)	0.063 (0.50)	**0.031 (0.58)**	0.070 (0.45)

TMJ: temporomandibular joint; TMD: temporomandibular disorders. * three item questions of the 3Q/TMD screening tool. Statistically significant differences are denoted in bold font.

**Table 3:** Results of the linear regression analyses examining predictors of perceived pediatric TMD knowledge, confidence in screening, diagnosis, and management.

Predictor	B	SE	*t*	*p* Value	95% CI
Perceived knowledge					
3Q-TMD	10.41	5.36	1.94	0.057	−0.33, 21.15
Years of practicing *					
0–2 years post-residency	−0.88	12.95	−0.07	0.946	−26.81, 25.05
2–5 years post-residency	−1.61	9.25	−0.17	0.863	−20.12, 16.91
5–10 years post-residency	−16.53	13.11	−1.26	0.212	−42.76, 9.71
10+ post-residency	15.87	6.03	2.63	0.011	3.81, 27.94
Adequacy of training	0.41	0.09	4.64	<0.001	0.23, 0.59
Frequency of pediatric patients seen	1.26	0.85	1.48	0.145	−0.45, 2.96
Confidence in screening for TMD					
3Q-TMD	5.87	6.28	0.94	0.345	−6.70, 18.44
Years of practicing *					
0–2 years post-residency	6.80	15.17	0.45	0.656	−23.56, 37.16
2–5 years post-residency	−8.97	10.83	−0.83	0.411	−30.65, 12.71
5–10 years post-residency	−18.36	15.35	−1.20	0.236	−49.08. 12.34
10+ post-residency	19.39	7.06	2.75	0.008	5.27, 33.51
Adequacy of training	0.47	0.10	4.59	<0.001	0.27, 0.68
Frequency of pediatric patients seen	1.61	1.00	1.61	0.112	−0.39, 3.60
Confidence in diagnosis of TMD					
3Q-TMD	8.56	6.17	1.39	0.171	−3.79, 20.92
Years of practicing *					
0–2 years post-residency	4.45	14.91	0.30	0.766	−25.39, 34.29
2–5 years post-residency	−3.50	10.65	−0.33	0.744	−24.81, 17.81
5–10 years post-residency	−26.55	15.09	−1.76	0.084	−56,74, 3.65
10+ post-residency	19.80	6.94	2.86	0.006	5.92, 33.68
Adequacy of training	0.43	0.10	4.22	<0.001	0.23, 0.63
Frequency of pediatric patients seen	1.33	0.98	1.35	0.181	−0.64, 3.29
Confidence in management of TMD					
3Q-TMD	5.04	6.71	0.75	0.456	−8.39, 18.47
Years of practicing *					
0–2 years post-residency	0.26	16.20	0.02	0.988	−32.18, 32.69
2–5 years post-residency	−2.07	11.57	−0.18	0.859	−25.23, 21.00
5–10 years post-residency	−20.13	16.40	−1.23	0.224	−52.95, 12.69
10+ post-residency	12.13	7.54	1.61	0.113	−22.96, 27.22
Adequacy of training	0.44	0.11	4.02	<0.001	0.22, 0.66
Frequency of pediatric patients seen	2.08	1.07	1.96	0.055	−0.05, 4.22

CI: confidence interval; SE: standard error. * reference: residents.

## Data Availability

The original contributions presented in the study are included in the article/Supplementary Materials; further inquiries can be directed to the corresponding author.

## Supplementary Materials

The following supporting information can be downloaded at: https://www.mdpi.com/article/10.3390/children13040445/s1, Supplementary Materials S1: Survey used in current study; Table S1: Perceived knowledge and confidence levels regarding TMD in pediatric populations by frequency of TMD assessment during screening history and clinical examination; Table S2: Perceived knowledge and confidence in screening, diagnosis, and management of pediatric TMD by years of clinical practice.

## Abbreviations

The following abbreviations are used in this manuscript:

AAO	American Association of Orthodontists
AEGD	Advanced education in general dentistry
ANOVA	Analysis of variance
CODA	Commission on Dental Accreditation
DC/TMD	Diagnostic Criteria for Temporomandibular Disorders
FQHC	Federally qualified health center
IRB	Institutional Review Board
TMJ	Temporomandibular joint
TMD	Temporomandibular disorder
3Q/TMD	Three screening questions for temporomandibular disorders
