# Supplementary material for: Orthodontists’ Perceived Knowledge, Confidence, and Clinical Practices in Pediatric Temporomandibular Disorders
Source: Children (Basel). 2026 Mar 25;13(4):445. doi: 10.3390/children13040445 (PMC13115295; doi:10.3390/children13040445)
Supplement: Supplementary file 1 [file children-13-00445-s001.zip › Supplemental Table s1.pdf]

Supplemental Materials Table s1. Perceived knowledge and confidence levels regarding TMD in pediatric populations by frequency of TMD assessment during screening history and clinical examination.

Screening assessment item	Frequency of screening assessment	Perceived knowledge about TMD	Confidence in screening for TMD	Confidence in diagnosis of TMD	Confidence in management of TMD	Sufficient training in TMD during orthodontic residency
		**Screening History**				
**Difficulty in mouth opening**	Always/most of the time/about half the time (n = 54)	64.0 ± 25.3	68.9 ± 28.3	66.7 ± 26.7	58.5 ± 28.9	56.1 ± 29.9
	Sometimes/never (n = 24)	41.5 ± 25.7	47.4 ± 31.4	44.2 ± 32.4	35.7 ± 31.3	39.4 ± 24.0
	***p* value (effect size)**	**<0.001 (0.88)**	**0.004 (0.74)**	**0.002 (0.79)**	**0.002 (0.77)**	**0.011 (0.59)**
**TMJ noise**	Always/most of the time/ about half the time (n = 58)	63.4 ± 26.6	69.3 ± 28.4	66.6 ± 28.0	59.5 ± 29.1	55.8 ± 29.7
	Sometimes/never (n = 20)	38.9 ± 21.0	41.8 ± 28.5	39.8 ± 28.0	28.4 ± 26.0	36.8 ± 22.7
	***p* value (effect size)**	**<0.001 (0.97)**	**<0.001 (0.97)**	**<0.001 (0.96)**	**<0.001 (1.10)**	**0.005 (0.68)**
**Pain in TMJ or pre-auricular area***	Always/most of the time/ about half the time (n = 54)	63.9 ± 25.6	69.1 ± 27.9	66.5 ± 27.4	59.4 ± 28.7	57.4 ± 29.0
	Sometimes/never (n = 24)	41.8 ± 25.3	47.0 ± 31.8	44.6 ± 31.3	33.7 ± 30.1	36.4 ± 24.0
	***p* value (effect size)**	**<0.001 (0.87)**	**0.003 (0.76)**	**0.003 (0.76)**	**<0.001 (0.88)**	**0.002 (0.76)**
**Pain with jaw function***	Always/most of the time/ about half the time (n = 55)	63.0 ± 25.1	68.6 ± 27.8	65.9 ± 27.1	56.8 ± 29.1	55.3 ± 28.8
	Sometimes/never (n = 23)	43.1 ± 27.9	47.1 ± 32.8	45.2 ± 32.8	38.8 ± 33.5	40.6 ± 27.7
	***p* value (effect size)**	**0.003 (0.77)**	**0.004 (0.73)**	**0.005 (0.72)**	**0.020 (0.59)**	**0.042 (0.51)**
**Change in bite**	Always/most of the time/ about half the time (n = 38)	67.0 ± 24.2	69.6 ± 27.8	69.9 ± 27.9	62.2 ± 28.8	54.5 ± 29.2
	Sometimes/never (n = 40)	47.7 ± 27.1	55.3 ± 32.1	52.1 ± 30.6	41.3 ± 30.6	47.6 ± 29.0

	***p* value (effect size)**	**0.001 (0.75)**	**0.038 (0.48)**	**0.020 (0.54)**	**0.003 (0.70)**	0.297 (0.24)
**Episodes of jaw lock***	Always/most of the time/ about half the time (n = 46)	62.5 ± 27.4	66.8 ± 30.4	64.9 ± 29.0	57.4 ± 30.8	53.7 ± 30.3
	Sometimes/never (n = 32)	49.4 ± 25.8	55.7 ± 30.5	52.3 ± 30.7	43.0 ± 30.6	47.0 ± 27.3
	***p* value (effect size)**	**0.037 (0.49)**	0.117 (0.37)[#]	0.069 (0.43)[#]	**0.046 (0.47)**	0.325 (0.23)
**Previous injuries to jaw, head, neck**	Always/most of the time/ about half the time (n = 59)	60.4 ± 26.4	65.8 ± 28.9	63.2 ± 28.6	55.8 ± 29.9	54.4 ± 28.8
	Sometimes/never (n = 19)	47.1 ± 28.7	51.4 ± 34.4	49.2 ± 33.3	38.2 ± 32.8	40.1 ± 28.0
	***p* value (effect size)**	0.065 (0.49)	0.075 (0.48)	0.078 (0.47)	**0.032 (0.58)**	0.062 (0.50)[#]
**Previous treatment for TMD**	Always/most of the time/ about half the time (n = 19)	72.8 ± 20.9	73.7 ± 26.4	73.7 ± 24.8	67.8 ± 27.9	56.9 ± 32.3
	Sometimes/never (n = 59)	52.1 ± 27.4	58.6 ± 31.3	55.3 ± 30.6	46.2 ± 30.8	49.0 ± 28.0
	***p* value (effect size)**	**0.003 (0.80)**	0.061 (0.50)	**0.019 (0.63)**	**0.008 (0.72)**	0.305 (0.27)
**Parafunctional activities**	Always/most of the time/ about half the time (n = 54)	60.5 ± 27.5	63.0 ± 30/7	61.5 ± 29.8	54.9 ± 31.5	51.2 ± 29.5
	Sometimes/never (n = 24)	49.5 ± 26.0	60.5 ± 31.5	55.8 ± 31.3	43.9 ± 30.2	50.3 ± 28.8
	***p* value (effect size)**	0.102 (0.41)	0.743 (0.08)	0.448 (0.19)	0.155 (0.35)	0.895 (0.03)
**Clinical Assessment**						
**Masticatory muscle palpation**	Always/most of the time/ about half the time (n = 53)	61.4 ± 25.2	67.8 ± 29.0	66.0 ± 27.0	57.5 ± 28.5	53.8 ± 30.1
	Sometimes/never (n = 25)	48.0 ± 29.9	50.6 ± 31.6	46.5 ± 32.7	38.7 ± 33.7	44.9 ± 26.5
	***p* value (effect size)**	**0.043 (0.50)**	**0.021 (0.57)**	0.007 (0.68)	0.012 (0.62)	0.211 (0.31)
**TMJ palpation**	Always/most of the time/ about	60.1 ± 25.7	66.7 ± 28.4	64.1 ± 27.5	55.6 ± 28.8	53.6 ± 29.4

	half the time (n = 59)					
	Sometimes/never (n = 19)	47.9 ± 31.0	48.5 ± 34.4	46.2 ± 34.8	38.6 ± 36.0	42.8 ± 27.3
	***p* value (effect size)**	0.093 (0.45)	**0.024 (0.61)**	**0.024 (0.61)**	**0.039 (0.56)**	0.162 (0.37)
**TMJ noise auscultation**	Always/most of the time/ about half the time (n = 29)	59.0 ± 28.9	64.3 ± 31.0	63.2 ± 31.5	57.4 ± 32.0	49.4 ± 30.2
	Sometimes/never (n = 49)	56.0 ± 26.7	61.0 ± 30.8	57.7 ± 29.6	48.0 ± 30.7	51.9 ± 28.7
	***p* value (effect size)**	0.643 (0.11)	0.649 (0.11)	0.446 (0.18)	0.204 (0.30)	0.717 (0.08)
**Mandibular range of motion**	Always/most of the time/ about half the time (n = 59)	60.0 ± 26.2	65.8 ± 29.7	63.3 ± 27.9	55.8 ± 28.7	53.9 ± 30.0
	Sometimes/never (n = 20)	48.9 ± 29.5	51.9 ± 32.0	49.6 ± 34.7	38.8 ± 35.8	42.3 ± 25.2
	***p* value (effect size)**	0.118 (0.41)	0.080 (0.46)	0.079 (0.46)	0.**035 (0.56)**	0.124 (0.40)

TMJ: temporomandibular joint; TMD: temporomandibular disorders.

*three item questions of the 3Q/TMD screening tool.

significance differed from the primary analysis when alternative frequency groupings were applied in sensitivity analyses.

Statistically significant differences are denoted in bold font.
