# Supplementary material for: Orthodontists’ Perceived Knowledge, Confidence, and Clinical Practices in Pediatric Temporomandibular Disorders
Source: Children (Basel). 2026 Mar 25;13(4):445. doi: 10.3390/children13040445 (PMC13115295; doi:10.3390/children13040445)
Supplement: Supplementary file 1 [file children-13-00445-s001.zip › Supplemental_Table s2.pdf]

Supplementary Materials Table s2. Perceived knowledge and confidence in screening, diagnosis, and management of pediatric TMD by years of clinical practice.

**Years of clinical practice**	**General knowledge about TMD**	**Screening of TMD**	**Diagnosis of TMD**	**Management of TMD**	**Sufficient training in TMD during orthodontic residency**
Currently in orthodontics residency	32.7 ± 19.1[a]	41.1 ± 31.4[a]	35.8 ± 25.2[a]	28.8 ± 18.2[a]	48.9 ± 21.1
0-2 years post-residency	55.5 ± 30.4	67.3 ± 33.4	62.5 ± 40.7	54.5 ± 35.2	52.5 ± 38.7
2-5 years post-residency	54.0 ± 23.0	46.0 ± 32.9	49.8 ± 23.7	43.8 ± 36.0	53.0 ± 35.3
5-10 years post-residency	28.3 ± 14.4[b]	27.6 ± 13.4[b]	22.6 ± 14.9[b]	21.4 ± 13.9[b]	34.7 ± 12.8
10+ years post-residency	66.5 ± 24.2[a,b]	72.1 ± 25.2[a,b]	70.2 ± 24.6[a,b]	59.6 ± 29.3[a,b]	53.1 ± 31.5
***p* value (effect size)**	**<.001 (0.31)**	**<.001 (0.29)**	**<.001 (0.34)**	**<.001 (0.22)**	.657 (0.03)

TMD: temporomandibular disorders.
Statistically significant differences are denoted in bold font.
