# Supplementary material for: Orthodontists’ Perceived Knowledge, Confidence, and Clinical Practices in Pediatric Temporomandibular Disorders
Source: Children (Basel). 2026 Mar 25;13(4):445. doi: 10.3390/children13040445 (PMC13115295; doi:10.3390/children13040445)
Supplement: Supplementary file 1 [file children-13-00445-s001.zip › Supplementary Materials 1.pdf]

Supplementary Materials 1. Survey Used in Current Study

Practice Patterns Among Orthodontists for the Management of Temporomandibular Disorders (TMD) in Pediatric Patients

Thanks for your interest in this project. The purpose of this survey is to evaluate practice patterns among orthodontists in the screening, diagnosis, and management of temporomandibular disorders (TMD) in pediatric patients to improve the management of TMD in children and adolescents.

Page Break

First, please answer a question to ensure that you're eligible.

Page Break

Are you currently completing or have already completed specialized training in orthodontics from a CODA-approved program in the United States?

- ○ Yes
- ○ No

Skip To: End of Survey If Are you currently completing or have already completed specialized training in orthodontics from... = No

Page Break

Great- you're eligible to complete the survey!

Page Break

For the first set of questions, please state your level of agreement with the following statements.

Page Break

Q1

Please state your agreement level (0=strongly disagree, 100=strongly agree).

	0 10 20 30 40 50 60 70 80 90 100
I am knowledgable about TMD in pediatric populations	
I received sufficient training in the screening, diagnosis, and management of TMD during my orthodontics residency	
I feel comfortable screening for TMD in pediatric patients	
I feel comfortable diagnosing TMD in pediatric patients	
I feel comfortable managing TMD in pediatric patients	

Page Break

For the next set of questions, please indicate the frequency of specific questions you ask and clinical examination items you use in practice.

Page Break

Q2 How often do you ask pediatric patients (and/or their parent) *“Do you have difficulty opening your mouth?”*

- Always
- Most of the time
- About half the time
- Sometimes
- Never

Q3 How often do you ask pediatric patients (and/or their parent) *“Do you hear noises within your jaw joint or in front of your ears?”*

- Always
- Most of the time
- About half the time
- Sometimes
- Never

Q4 How often do you ask pediatric patients (and/or their parent) *“Do you have pain in or around your ears or your cheeks?”*

- Always
- Most of the time
- About half the time
- Sometimes
- Never

Q5 How often do you ask pediatric patients (and/or their parent) *“Do you have pain when chewing, talking, opening wide, or using your jaw?”*

- Always
- Most of the time
- About half the time
- Sometimes
- Never

Q6 How often do you ask pediatric patients (and/or their parent) *“Has your bite felt uncomfortable or unusual?”*

- Always
- Most of the time
- About half the time
- Sometimes
- Never

Q7 How often do you ask pediatric patients (and/or their parent) *“Does your jaw ever lock or go out of place?”*

- Always
- Most of the time
- About half the time
- Sometimes
- Never

Q8 How often do you ask pediatric patients (and/or their parent) *“Have you ever had an injury to your jaw, head, or neck?”*

- Always
- Most of the time
- About half the time
- Sometimes
- Never

Q9 How often do you ask pediatric patients (and/or their parent) *“Have you previously been treated for temporomandibular disorders?"*

- ◯ Always
- ◯ Most of the time
- ◯ About half the time
- ◯ Sometimes
- ◯ Never

Q10 How often do you ask pediatric patients (and/or their parent) *“Do you grind or clench your teeth during the day or at night?”*

- ◯ Always
- ◯ Most of the time
- ◯ About half the time
- ◯ Sometimes
- ◯ Never

Q11 During clinical exam, how often do you palpate the muscles of mastication for tenderness, pain, or pain referral patterns in pediatric patients?

- ◯ Always
- ◯ Most of the time
- ◯ About half the time
- ◯ Sometimes
- ◯ Never

Q12 During clinical exam, how often do you palpate the TMJ for pain and/or tenderness in pediatric patients?

- ◯ Always
- ◯ Most of the time
- ◯ About half the time
- ◯ Sometimes
- ◯ Never

Q13 During clinical exam, how often do you auscultate for TMJ sounds in pediatric patients?

- ◯ Always
- ◯ Most of the time
- ◯ About half the time
- ◯ Sometimes
- ◯ Never

Q14 During clinical exam, how often do you assess for mandibular range of motion in pediatric patients?

- ◯ Always
- ◯ Most of the time
- ◯ About half the time
- ◯ Sometimes
- ◯ Never

Page Break

For the next set of questions, please indicate your current clinical encounters.

Q15 Which malocclusion do you feel like is most correlated with TMD in pediatric patients? *Check all that apply.*

- ☐ Anterior crossbite
- ☐ Posterior crossbite
- ☐ Increased overjet
- ☐ Increased overbite
- ☐ Open bite
- ☐ Skeletal class malocclusion
- ☐ Dental class malocclusion
- ☐ Overcrowding
- ☐ Presence of spacing
- ☐ Skeletal hyperdivergency
- ☐ Skeletal hypodivergency
- ☐ Other; please describe ____

Q16 Approximately what percentage of pediatric patients are referred to you for TMD?

- ◯ 0%
- ◯ Up to 25%
- ◯ Up to 50%
- ◯ Up to 75%
- ◯ Almost 100%

Q17 For pediatric patients who are referred to you for TMD, where are they most often referred from? *Check all that apply.*

- ▢ Pediatric dentist
- ▢ General dentist
- ▢ Oral and maxillofacial surgeon
- ▢ Orofacial pain specialist
- ▢ Speech therapist
- ▢ Chiropractor
- ▢ Physical therapist
- ▢ I don't receive any pediatric patients referred for TMD.
- ▢ Other; please describe ____

Page Break

Q18 On average, what percentage of pediatric patients do you see presenting with TMD signs and/or symptoms <u>per week</u>?

○ 0%

○ Up to 25%

○ Up to 50%

○ Up to 75%

○ Almost 100%

○ I don’t routinely assess for TMD signs and/or symptoms in pediatric patients.

Q19 What is the predominant pediatric age group that you see presenting with signs and/or symptoms of TMD? *Check all that apply.*

☐ 5 years or younger

☐ 6 to 8 years

☐ 9 to 11 years

☐ 12 to 14 years

☐ 15 to 18 years

☐ None of my pediatric patients present with signs and/or symptoms of TMD.

☐ I don't routinely assess for TMD signs and/or symptoms in pediatric patients.

Q20 When you have a patient presenting with signs and/or symptoms of TMD, what treatment do you recommend? *Check all that apply.*

- ☐ Stop or defer orthodontic treatment
- ☐ Fabricate occlusal splint
- ☐ Perform occlusal adjustment
- ☐ Refer to specialist
- ☐ Patient education
- ☐ Physical therapy (e.g., jaw exercises, massage)
- ☐ Behavioral therapy (e.g., biofeedback, relaxation training, stress management, etc.)
- ☐ Prescription medication (e.g., non-steroidal anti-inflammatory drugs, anxiolytic agents, muscle relaxants, etc.)
- ☐ Order TMJ imaging assessment (e.g., panoramic radiograph, computed tomography, magnetic resonance imaging, etc.)
- ☐ None of my pediatric patients present with signs and/or symptoms of TMD.
- ☐ I don't routinely assess for TMD signs and/or symptoms in pediatric patients.

Display This Question:

If When you have a patient presenting with signs and/or symptoms of TMD, what treatment do you recom... = Refer to specialist

Q21 Which specialist(s) do you most often refer to when a pediatric patient presents with signs and/or symptoms with TMD? *Check all that apply.*

- ☐ Pediatric dentist
- ☐ Orofacial pain specialist
- ☐ Oral and maxillofacial surgeon
- ☐ Primary care physician
- ☐ Psychologist
- ☐ Physical therapist
- ☐ Other; please describe ____

Q22 Which co-morbidities do you most commonly encounter in pediatric patients presenting with TMD signs and/or symptoms? *Check all that apply.*

- ☐ Sleep disorders
- ☐ Arthritis
- ☐ Irritable bowel syndrome
- ☐ Headache/migraine
- ☐ Allergies
- ☐ Anxiety
- ☐ Depression
- ☐ Behavioral/developmental disorders (e.g., autism spectrum disorder, attention-deficit/hyperactivity disorder)
- ☐ Other; please describe ____
- ☐ None of my pediatric patients present with signs and/or symptoms of TMD.
- ☐ I don't routinely assess for TMD signs and/or symptoms in pediatric patients.

Page Break

For the next set of questions, please state your level of agreement with the following statements.

Q23

Please state your agreement level (0=strongly disagree, 100=strongly agree).

	0 10 20 30 40 50 60 70 80 90 100
Orthodontic treatment causes TMD	
Orthodontic treatment can treat TMD	
Orthodontic treatment has no relationship with TMD	
Malocclusion is one of the contributing factors to TMD	

Page Break

These last few questions ask about you and your training.

Page Break

Q24 Besides orthodontics residency, what additional training have you received that is specifically relevant to TMD?

- ☐ Additional postgraduate specialty training
- ☐ Continuing education courses
- ☐ Simulation training
- ☐ Attendance at national and/or international conferences
- ☐ Other; please describe ____
- ☐ None

Q25 Which of these educational resources would be most helpful for you as an orthodontist working with patients presenting with TMD signs and/or symptoms? *Check all that apply.*

- ☐ More studies to determine the prevalence of TMD and its clinical implications for practice
- ☐ Continuing education courses and training focused on the management of TMD
- ☐ Clearer guidance from national societies for the screening, diagnosis, and management of TMD
- ☐ Other; please describe ____

Q26 How many years have you been practicing orthodontics?

- ◯ Currently in orthodontics residency
- ◯ 0-2 years post residency
- ◯ 2-5 years post residency
- ◯ 5-10 years post residency
- ◯ 10+ years post residency

Q27 How many days per week do you practice or encounter orthodontics?

- ◯ 0
- ◯ 1
- ◯ 2
- ◯ 3
- ◯ 4
- ◯ 5
- ◯ 6+

Q28 What type of clinical setting do you primarily work in?

- ○ Community clinic or federally qualified health center
- ○ Private practice
- ○ Hospital
- ○ Academic institution
- ○ Other; please describe ____

Q29 Which region do you primarily practice in?

- ○ Northeast
- ○ Midwest
- ○ West
- ○ Southeast
- ○ Southwest

Q30 In addition to orthodontics, what other dental training have you received? *Check all that apply*.

- [ ] General Practice Residency
- [ ] Advanced Education in General Dentistry
- [ ] Oral Medicine
- [ ] Orofacial Pain
- [ ] Dental Anesthesiology
- [ ] Oral and Maxillofacial Surgery
- [ ] Oral and Maxillofacial Pathology
- [ ] Pediatric Dentistry
- [ ] Endodontics
- [ ] Prosthodontics
- [ ] Periodontics
- [ ] Dental Public Health
- [ ] Other; please describe ____
- [ ] None

Q31 What is your age?

____

Q32 Which gender do you identify with?

- ◯ Male
- ◯ Female
- ◯ Non-binary / third gender
- ◯ Prefer not to answer

Q33 Which ethnicity do you identify with?

- ◯ Hispanic or Latinx
- ◯ Not Hispanic or Latinx
- ◯ Unsure
- ◯ Prefer not to answer

Q34 What race do you identify with?

- ◯ White
- ◯ Black or African American
- ◯ American Indian or Alaska Native
- ◯ Asian
- ◯ Native Hawaiian or Other Pacific Islander
- ◯ Other
- ◯ Unsure
- ◯ Prefer not to answer

Page Break

Please share any other thoughts or feedback related to managing TMD in pediatric dental patients from an orthodontist's perspective.

____

Page Break

End of Block: Default Question Block
